# Molecular identification and biological characterization of *Cryptosporidium muris* from a naturally infected cat (*Felis catus*)

**DOI:** 10.1017/S0031182026102741

**Published:** 2026-08

**Authors:** Jana Fenclová, Nikola Holubová, Lenka Hlásková, Magdalena Szydłowicz, Bohumil Sak, Martin Kváč

**Affiliations:** 1 Faculty of Agriculture and Technology, University of South Bohemia in České Budějovice, České Budějovice, Czech Republic; 2 Institute of Parasitology, Biology Centre of the Academy of Sciences of the Czech Republic, v.v.i., České Budějovice, Czech Republic; 3 Department of Biology and Medical Parasitology, Wroclaw Medical University, Wroclaw, Poland

**Keywords:** cat, *Cryptosporidium muris*, mice infection, multilocus genotyping, oocyst degradation

## Abstract

*Cryptosporidium muris* is a gastric species primarily infecting rodents, whereas natural infections in cats are rarely documented and biologically poorly characterized. Oocysts of *C. muris* were detected in a cat and monitored for 120 days. Molecular characterization was performed on the small subunit rRNA, actin, 70-kDa heat shock protein*, Cryptosporidium oocyst* wall protein and thrombospondin-related adhesive protein of *Cryptosporidium*-1 loci. Oocyst morphology was evaluated microscopically. Infectivity and shedding dynamics were assessed in BALB/c mice and compared with *C. muris* RN66 and *C. proliferans*. Oocyst integrity during storage at 4°C was monitored for 60 days at 15-day intervals using flow cytometry and viability assays. The infected cat shed oocysts for 97 days, with a maximum intensity of 371 200 oocysts per gram (OPG), yet remained clinically asymptomatic. Developmental stages were confirmed histologically in gastric biopsy specimens. Haematological and biochemical parameters remained within reference ranges, and no concurrent parasitic, bacterial or viral infections were detected; only seropositivity to *Toxoplasma gondii* was recorded. All analysed loci showed 100% identity with *C. muris* RN66. Oocyst morphology did not differ from reference isolates. However, oocysts originating from the cat exhibited markedly reduced environmental stability, with a 90% decline in integrity within 60 days. Experimental infection of mice confirmed infectivity, but total oocyst output (32 300 OPG/days) was several orders of magnitude lower than that of *C. muris* RN66. Although genetically identical to rodent isolates, the feline-derived *C. muris* displayed altered biological properties, including reduced oocyst stability and transmission fitness. Cats may serve as susceptible but epidemiologically inefficient hosts.

## Graphical abstract

**Figure:**
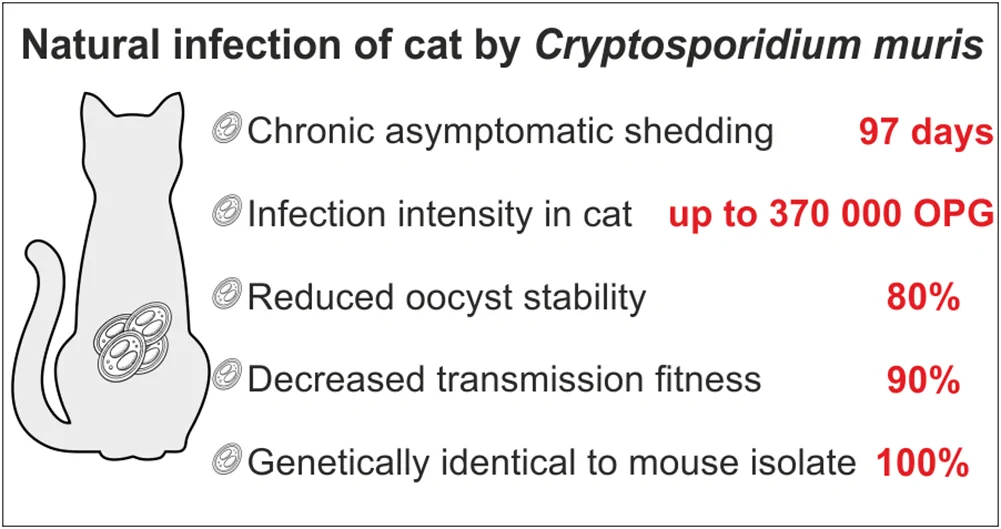

## Introduction

Protozoa of the genus *Cryptosporidium* are epicellular parasites that infect the gastrointestinal and respiratory tracts of a wide range of vertebrates (O’Donoghue, 1995). Currently, 48 species of *Cryptosporidium* and a large number of genotypes that differ in tissue and host specificity have been validly described (Zikmundová et al., 2025). Within the genus *Cryptosporidium*, we distinguish 2 phylogenetic groups: species that infect the stomach of their hosts and species that infect the intestine, whereby within this group we can further distinguish species and genotypes that parasitize the small intestine, caecum, large intestine or cloaca (Fayer and Xiao, 1997). In terms of host specificity, most species and genotypes are narrowly host-specific, infecting only a limited number of phylogenetically closely related hosts (Ryan et al., 2014; Feng et al., 2018). The second, less numerous group, comprising mainly *C. parvum*, the species with the widest host range, as well as *C. ubiquitum* and *C. baileyi*, represents cryptosporidia infectious to a wide range of hosts across different orders of mammals or birds.

This group also includes *C. muris*, a species that primarily parasitizes rodents (Iseki et al., 1989; Aydin and Ozkul, 1996; Chalmers et al., 1997; Torres et al., 2000), but which has also been detected in humans, wood mice, bank voles, Syrian hamsters, desert hamsters, squirrels, Siberian chipmunks, marsupials, Bactrian camels, reticulated giraffe, mountain goats, lambs, rabbits, ringed seals, hyraxes, cynomolgus monkeys, dogs and cats (Iseki et al., 1989; Aydin and Ozkul, 1996; Katsumata et al., 2000; Hůrková et al., 2003; Kváč et al., 2016).

Cats are among the hosts in which infection has been documented under both experimental and natural conditions. Iseki et al. demonstrated that *C. muris* is infectious to cats and causes long-lasting and massive infection (Iseki et al., 1989). Although it could be assumed that *C. muris* infection in cats should be common, given that rodents – the natural hosts of *C. muris* (Tyzzer, 1910; Xiao et al., 2004) – are their typical prey, the number of described natural cases of infection in cats is very low (Table 1). To date, the detailed biological characteristics of *C. muris* isolates obtained from cats remain unknown. Most studies have determined the species based on partial sequences of the small subunit *(SSU)* rRNA (Ryan et al., 2021). Only Pavlásek and Ryan (2007) demonstrated the infectivity of an isolate from a cat to mice, but without further details. This study aims to describe the course of chronic, natural infection of cats with *Cryptosporidium muris*, including molecular genotyping of the isolate and a description of its biological properties, including oocyst morphology, infectivity for model hosts and oocyst viability.

**Table 1.** Overview of published reports of *Cryptosporidium muris* infection in cats Table 1 long description.

Year of detection	Type of infection	Number of cats examined	Diagnostic method	Evidence of oocyst infectivity	Sampling strategy	Reference
1989	Experimental infection	3	Microscopy	Yes (mice)	Repeated sampling	(Iseki et al., 1989)
2006	Natural infection	1	PCR	No	One-shot sampling	(Santín et al., 2006)
2007		1	Microscopy, PCR	Yes (mice)	Repeated sampling	(Pavlásek and Ryan, 2007)
2011		1	Histology, PCR	No	Repeated sampling	(FitzGerald et al., 2011)
2015		1	PCR	No	One-shot sampling	(Yang et al., 2015)
2025		1	PCR	No	One-shot sampling	(Barrios et al., 2025)

## Materials and methods

### Source and collection of specimens

As part of routine parasitological screening at a veterinary clinic, 5 domestic cats (*Felis catus* f. domestica) aged 1–6 years, originating from a single breeder, were examined. Routine screening included coprological examination by flotation with Sheather’s sugar solution to detect helminth eggs and protozoan oocysts, and formalin–ethyl acetate sedimentation to detect *Giardia* cysts. In cases of suspected cryptosporidiosis or giardiasis, a commercially available immunochromatographic rapid test for *Cryptosporidium* and *Giardia* (R-Biopharm, Darmstadt, Germany) was also used. During this routine screening, *Cryptosporidium* spp. oocysts morphologically consistent with *C. muris* were detected in 1 cat. This cat subsequently underwent abdominal ultrasonography focused on the gastrointestinal tract and gastroscopy of the stomach. Faecal samples and gastric biopsy specimens were submitted for further parasitological examination and genotyping to the Institute of Parasitology, Biology Centre of the Czech Academy of Sciences (BC CAS), Czech Republic.

After species identification as *C. muris*, all cats were monitored regularly for the presence of oocysts and *C. muris*–specific DNA. For this targeted follow-up monitoring, faecal smears were stained with aniline–carbol–methyl violet, according to previously described protocol (Miláček and Vítovec, 1985). Subsequently, oocysts were additionally examined to verify and compare staining characteristics using a modified Ziehl–Neelsen method (Henriksen and Pohlenz, 1981) and auramine–phenol staining (Casemore, 1991). In addition, FITC-conjugated *Cryptosporidium*-specific antibodies targeting the oocyst wall were used (IFA; *Cryptosporidium* IF Test, Cryptocel, Cellabs Pty Ltd., Brookvale, Australia).

### Molecular analyses

Total genomic DNA (gDNA) was extracted from 200 mg of faeces using the Exgene™ Stool DNA mini kit (GeneAll Biotechnology Co Ltd., Seoul, Korea). The purified DNA was stored at –20°C until further processing. Partial sequences of the genes encoding the *SSU* rRNA, actin, the *Cryptosporidium* oocyst wall protein (*COWP*), the 70-kDa heat shock protein (*HSP70*) and the thrombospondin-related adhesive protein of *Cryptosporidium*-1 (*TRAP-C1*) were amplified using previously published nested PCR protocols and primer sets (Xiao et al., 1999; Pedraza-diaz et al., 2001; Sulaiman et al., 2002; Jiang et al., 2005; Kváč et al., 2016). Both primary and secondary PCR reactions were performed in 50 μL reaction containing 2 μL of gDNA template (or 2 μL of primary template in case of secondary reaction), 2.5 U DreamTaq Green DNA Polymerase (Thermo Fisher Scientific, Waltham, MA, USA), 1 × PCR buffer, 200 nM of each primer, 200 μM of each dNTP, 2 μL of non-acetylated bovine serum albumin (BSA; 10 mg/mL; New England Biolabs, Beverly, MA, USA) and molecular grade water. gDNA from *Cryptosporidium*-negative faecal samples were used as negative controls and *C. serpentis* DNA as a positive control. PCR products were separated on 2% agarose gels stained with ethidium bromide, purified using the Monarch ® DNA Gel Extraction Kit (New England Biolabs, Ipswich, MA, USA) and sequenced in a commercial laboratory (SeqMe, s.r.o., Dobřiš, Czech Republic) using secondary primers. The identity of the isolates was verified by BLAST analysis using the GenBank database.

### qPCR-infection intensity

*Cryptosporidium muris* infection was quantified using a TaqMan-based qPCR assay and previously published protocols with the following modification (Hill et al., 2007). Briefly, each 20 µL reaction contained 2 µL gDNA, 400 nM forward (5´-ATGACGGGTAACGGGGAAT-3´) and reverse primers (5´-CCCTGTGTCAGGATTGGGTA-3´), 200 nM 5´ FAM and 3´BHQ1-labelled TaqMan probe (5´-CGCGCCTGCTGCCTTCCTTAGATG-3´) and 10 µL Luna Universal Probe qPCR master Mix (New England BioLabs Inc., Ipswich, MA, USA). A standard (calibration) curve was constructed using serial dilution of DNA isolated from 10^7^ *Cryptosporidium proliferans* oocysts. Infection intensity was expressed as the number of oocysts per gram (OPG) of faeces.

### Health monitoring of cats

Cats underwent baseline clinical laboratory screening that included haematological and biochemical evaluation of peripheral blood. A complete blood count with leukocyte differential was performed, reporting total leukocytes (WBC) and relative and absolute leukocyte subsets (neutrophils, lymphocytes, monocytes, eosinophils, basophils). Erythrocyte variables and indices (RBC, haemoglobin, haematocrit, MCV, MCH, MCHC, RDW) and platelet variables and indices (PLT, MPV, PDW, PCT, P-LCR/P-LCC) were also recorded, including the presence/quantity of nucleated red blood cells (NRBCs) when reported by the analyser. Serum/plasma biochemistry comprised total protein, albumin, total bilirubin, AST (GOT), ALT (GPT) and LDH activities, urea (BUN), glucose and creatinine. In addition, total thyroxine (T4; v-T4) was measured. All results were interpreted against laboratory-specific reference intervals. In addition, cats were screened for major feline infectious diseases, including feline leukaemia virus, feline immunodeficiency virus, feline coronavirus, presence of gastrointestinal parasites and *Toxoplasma gondii* antibodies. All animals were clinically monitored daily for activity level, noticeable body-weight loss, appetite and the frequency and consistency of faeces.

### Histological examination

Gastric biopsy samples were fixed in 10% neutral buffered formalin and subsequently processed using standard histological procedures. Histological sections (5 μm thick) were stained with haematoxylin and eosin and periodic acid-Schiff (PAS) and examined under 100–400 × magnification using an Olympus IX70 microscope (Kváč and Vítovec, 2003).

### Oocyst morphology

For morphological analysis and experimental infection, *C. muris* oocysts were purified from feline faeces by sucrose and caesium chloride gradient centrifugation (Arrowood and Sterling, 1987; Kilani and Sekla, 1987) and stored in dH_2_O without antibiotics and antimycotics at 4°C. Morphometric measurements were performed using differential interference contrast (DIC) microscopy at 1000 × magnification (Olympus BX51, Olympus Corporation, Tokyo, Japan). Photomicrographs used for morphometric analysis were evaluated with Olympus CellSens Entry 2.1 software in combination with an Olympus DP73 digital colour camera (Olympus Corporation). The length and width of 100 oocysts were measured, and a shape index was calculated.

### Experimental infection and oocyst infectivity

Oocysts of *C. muris* RN66 (Waterborne Inc.; New Orleans, LA, USA), a laboratory reference isolate originally obtained from *Rattus norvegicus* in Japan (Iseki, 1986), and *C. proliferans* (Biology Centre of the Czech Academy of Sciences (CAS), Czech Republic), purified and stored similarly as oocysts of *C. muris* CAT21, the feline isolate characterized in the present study, were used as controls. Infectivity for BALB/c mice and the course of infection were evaluated using oocysts immediately after purification and those stored for no more than 5 days. In addition, aliquots of oocysts of individual species and strains were stored for 6 months, and their viability was assessed monthly using the FDA/PI assay (Jones and Senft, 1985), together with testing of infectivity in model hosts and monitoring of natural excystation and oocyst loss (see below).

For each species/strain of *Cryptosporidium*, six 6-week-old BALB/c mice (3 males and 3 females) were inoculated with 1 000 000 oocysts of the respective isolate suspended in 200 µL of water. Six age- and sex-matched control mice (3 males and 3 females) were inoculated with 200 µL of water only. From the 5th day post-infection (DPI), individual faecal samples were collected and examined for oocyst shedding and *Cryptosporidium*-specific DNA. Each sample was processed for microscopy (aniline–carbol–methyl violet staining) and qPCR (see above). In groups infected with oocysts stored for ≤5 days after isolation, mice were monitored for 30 days, and the individual infection intensity was evaluated. In mice infected with oocysts stored for 1–6 months, only the presence of infection was assessed at 20 DPI. If no oocysts or specific DNA were detected within 20 DPI in mice infected with 1–6-month-old oocysts, the animals were necropsied, and stomach tissue was analysed for the presence of *Cryptosporidium*-specific DNA/qPCR. From each animal that tested positive for *Cryptosporidium* during the experiment, genotyping of the isolate was performed to confirm the identity with the inoculum.

### Animal care

Mice were housed under standard conditions in separate plastic cages in the animal facility of the Institute of Parasitology, Biology Centre of the Czech Academy of Sciences (BC CAS), v.v.i., in České Budějovice, Czech Republic. The light–dark cycle was set to 12 h light and 12 h dark at 22°C and 50–55% relative humidity. The mice were fed a commercial pelleted rodent diet (Altromin 1314) and provided water *ad libitum*. All samples were collected by animal breeders in accordance with Czech legislation (Act No. 246/1992 Coll. on the Protection of Animals Against Cruelty) and relevant European regulations. The study design was reviewed and approved by the Ethics Committee of the Biology Centre of the Czech Academy of Sciences (protocol No. 73-2024-P).

### Flow cytometry analysis

To assess temporal changes in *C. muris* CAT21 oocyst counts (degradation), 20 mL of purified oocysts (2 × 10^6^ oocysts/mL) was aliquoted into 50 mL screw-cap tube. The same procedure was performed for *C. proliferans* and *C. muris* RN66 oocysts used as controls. Suspensions were stored at 4°C and analysed on days 0, 15, 30, 45 and 60. Prior to acquisition, oocyst suspensions were vortexed thoroughly and 450 μL (0.45 × 10^6^ oocysts) was withdrawn from each stock in 5 technical replicates. Each replicate was spiked with 50 μL fluorescent counting beads (Flow-Count Fluorospheres; Beckman Coulter, USA). Absolute oocyst numbers were determined by single-platform bead-based flow cytometry on a BD FACSCanto II using BD FACSDiva software. Beads were identified as a discrete high-fluorescence population and used for normalization of event counts, as described for bead-enhanced absolute quantitation. Oocysts were enumerated without antibody staining using a morphology-driven gating strategy based on forward and side scatter (FSC-A/SSC-A) to discriminate oocysts from residual debris, followed by a second refinement gate to exclude remaining contaminants, consistent with published protocols for unstained *Cryptosporidium* oocyst quantification. Doublets were excluded using standard area-versus-height (or width) discrimination (Sonzogni-desautels et al., 2019). Absolute oocyst concentration was calculated from the oocyst-to-bead event ratio and the manufacturer-assigned bead concentration (or bead number per test volume), and results were expressed relative to day 0 and to the control species to estimate the proportion of degraded oocysts over time.

### Statistical analyses

Differences in oocyst size of *C. muris* CAT21 obtained from different hosts, as well as differences between oocysts of *C. muris* CAT21 and those of other *C. muris* isolates and *C. proliferans*, were tested using Hotelling’s multivariate 2-sample *t*-test implemented in the ICSNP (Tools for Multivariate Nonparametrics) package in R version 4.2.2. The total oocyst output was quantified as the area under the oocyst shedding curve (AUC), calculated for each animal using the trapezoidal rule based on daily oocyst counts (OPG) over the 30-day observation period. AUC values were compared among isolates (*C. muris* CAT21, *C. muris* RN66 and *C. proliferans*) using one-way analysis of variance (ANOVA). When significant differences were detected, pairwise comparisons were performed using Tukey’s honestly significant difference (HSD) post hoc test. All statistical analyses were performed in R version 4.2.2 (R Core Team, 2019). A *P*-value < 0.05 was considered statistically significant.

## Results

### Detection, genotyping and quantification of C. muris in cats

**Figure 1.**
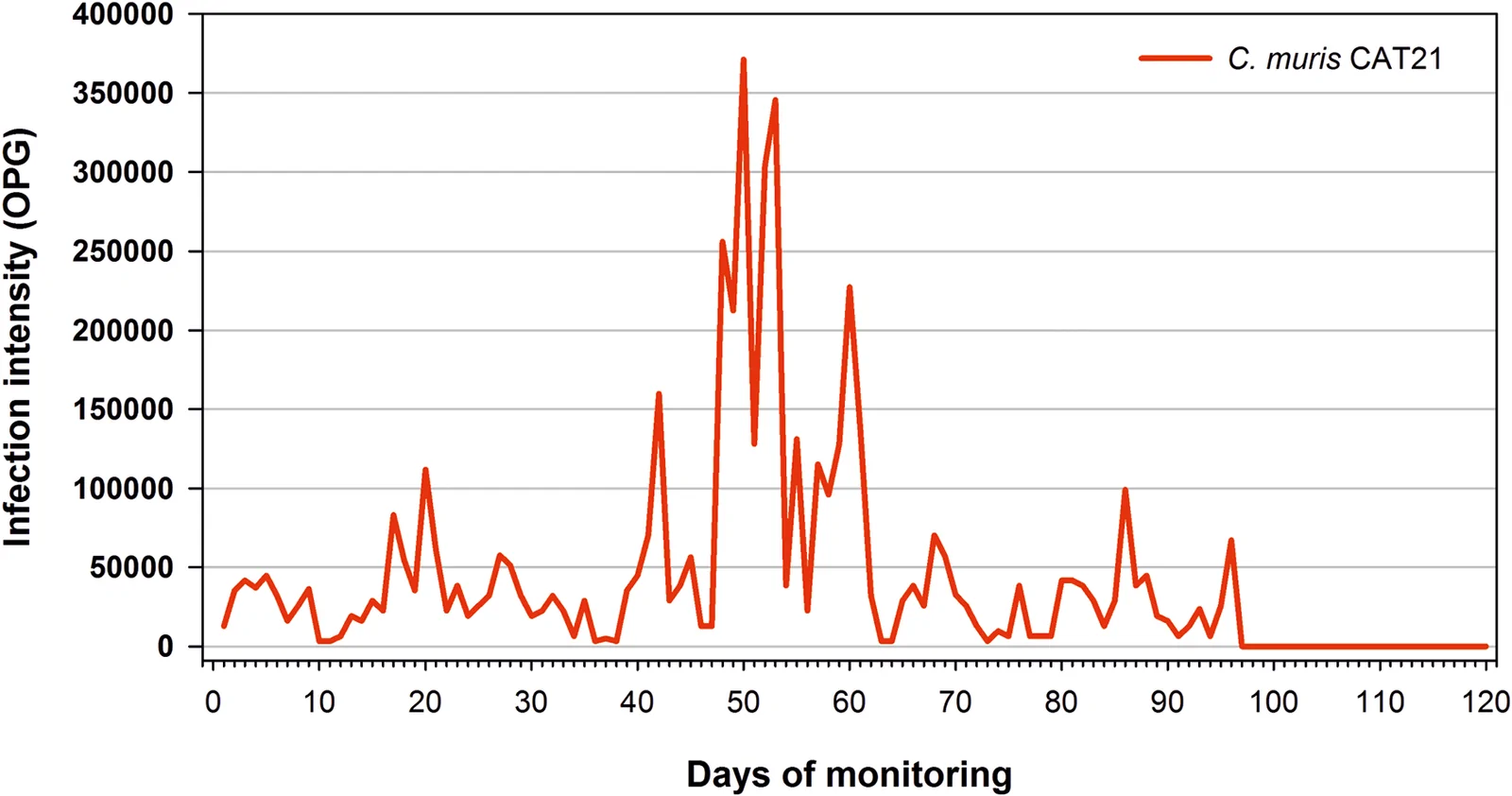
Dynamics of *C. muris* CAT21 oocyst shedding in a naturally infected domestic cat during a 120-day observation period.

During routine parasitological examination, oocysts of *Cryptosporidium* sp., morphologically consistent with gastric species of mammalian cryptosporidia, were detected in 1 of 5 cats originating from the same breeder (cat no. 4). At the initial examination, the infection intensity was 12 800 OPG, and subsequent *SSU* molecular analysis confirmed the presence of *C. muris* DNA (100% identical to sequence GenBank Acc. No. AB089284). All animals were subsequently re-examined. With the exception of cat no. 4, in which *C. muris* infection was confirmed at the following sampling (35 200 OPG), all other cats were negative for *Cryptosporidium*. Owing to the repeated faecal positivity and the high infection intensity, the cats were monitored regularly. The isolate was designated *C. muris* CAT21. During the 120-day observation period, regular shedding of *C. muris* CAT21 was recorded in cat no. 4, with a maximum infection intensity of 371 200 OPG (Figure 1). This cat shed oocysts for 97 days following the first detection. No other cat living in the same household was positive for *Cryptosporidium*. Molecular analyses of the *SSU* (AB089284), actin (XM002140165), *HSP70* (XM002140816), *COWP* (DQ060430) and *TRAP-C1* (XM002142580) genes demonstrated 100% sequence identity of the CAT21 isolate with *C. muris* RN66.

### Health status

Throughout the observation period, no symptoms of clinical cryptosporidiosis were detected in the naturally infected domestic cat. The animal did not suffer from diarrhoea or vomiting, and no other gastrointestinal problems were observed. The animal ate normally, and its weight was appropriate for its age, sex, feed intake and activity level. Similar to the cat, we did not observe any clinical symptoms associated with *C. muris* CAT21 infection in experimentally infected BALB/c mice. In the baseline laboratory screening, most haematological and biochemical variables of the cat were within the respective laboratory reference intervals and were not indicative of marked anaemia, thrombocytopenia, or overt renal/hepatocellular dysfunction. Total leukocyte counts ranged from 15.88 to 22.11 × 10^9^/L, with mild leukocytosis and neutrophilia observed in 1 sample (WBC 22.11 × 10^9^/L; NEU# 14.924 × 10^9^/L). Erythrocyte parameters were generally stable (e.g. HCT 40.6–51.2%, with a single mildly increased value of 51.2%), and platelet counts were within the provided reference range (PLT 225–416 × 10^9^/L). NRBCs were flagged by the analyser in several samples (NRBC# 7.77–44.30 × 10^9^/L) and were interpreted in conjunction with the overall haematological profile. Biochemistry showed values consistent with adequate renal function (creatinine 53–88 µmol/L; urea 8.5–13.3 mmol/L) and no relevant hyperbilirubinaemia (total bilirubin 5–6 µmol/L). Total thyroxine concentrations were within the laboratory reference range (v-T4 22.86–28.95 nmol/L; reference 11.6–47.6 nmol/L).

### Gastroscopy

**Figure 2.**
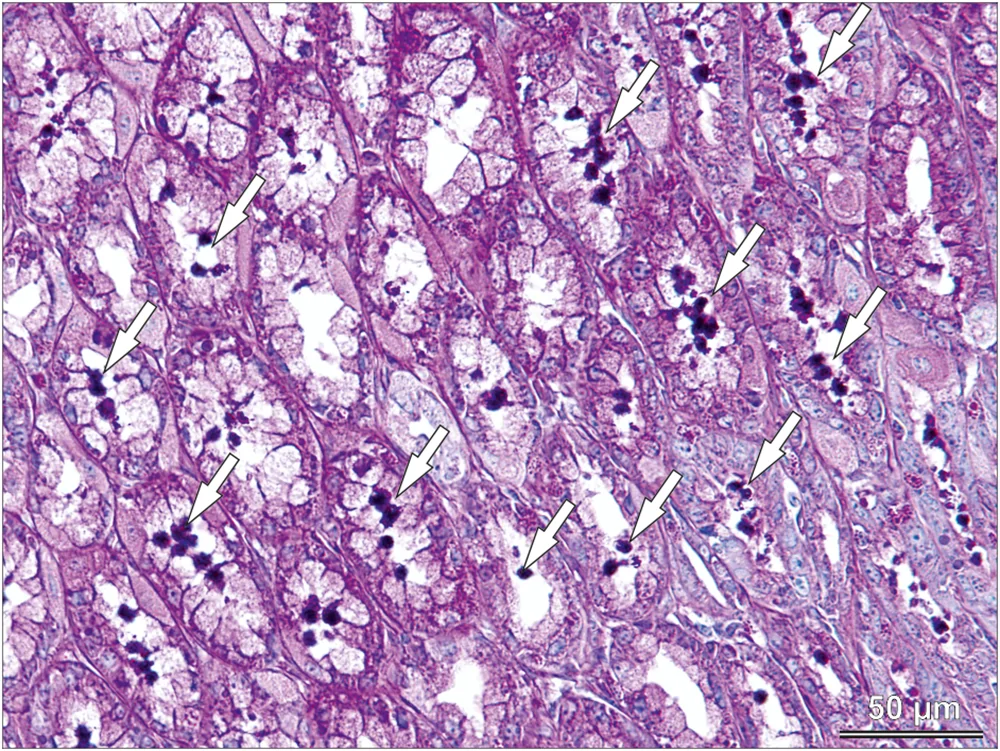
Histological section of a gastric biopsy demonstrating developmental stages of *C. muris* CAT21 in a naturally infected domestic cat (PAS stain, 400 × magnification). Arrows: Developmental stages of *C. muris* CAT21.

Gastroscopy performed on day 45 of the observation period revealed areas of evident gastric mucosal oedema localized predominantly in the *pyloric antrum*. Tissue samples were collected from these regions for histological examination, which demonstrated the presence of developmental stages of *Cryptosporidium* (Figure 2).

### Oocyst morphology

**Figure 3.**
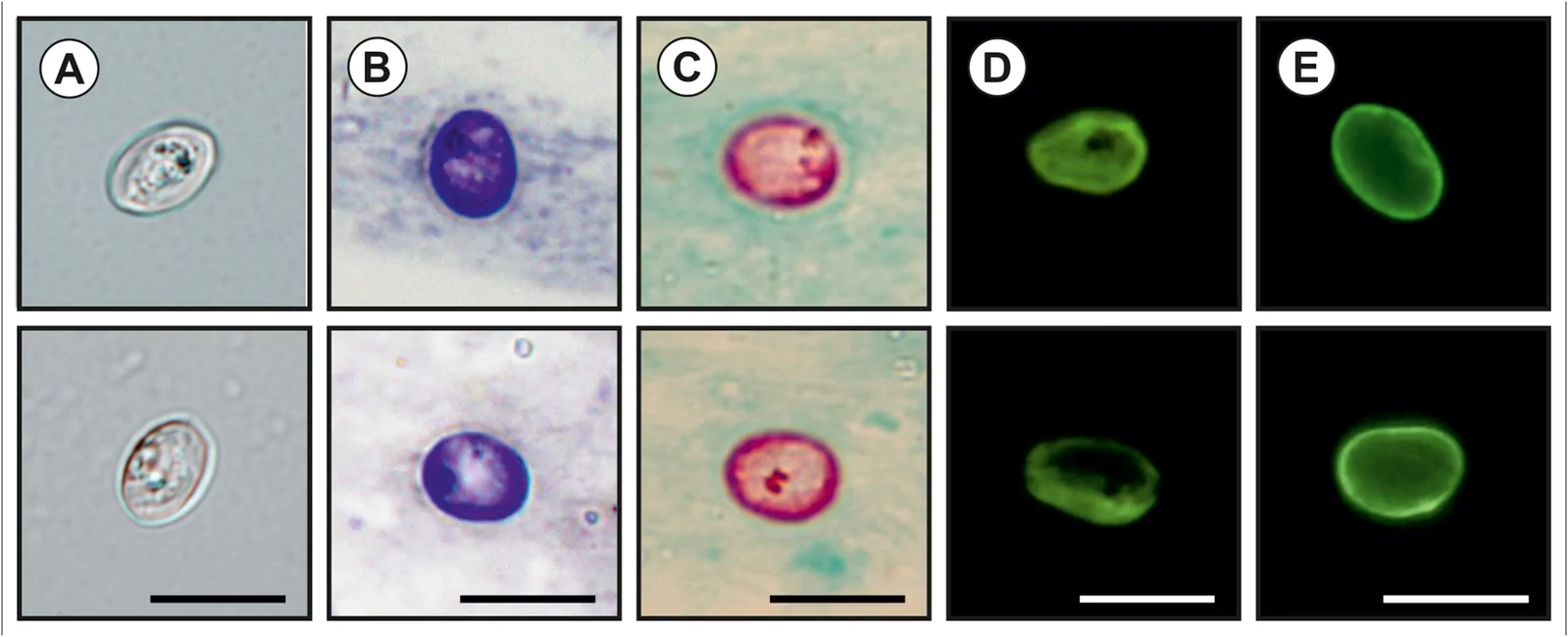
Oocysts of *Cryptosporidium muris* CAT21 (A) in differential interference contrast microscopy, (B) stained by aniline–carbol–methyl violet staining, (C) stained by Ziehl–Neelsen staining, (D) stained by auramine-phenol staining and (E) labelled with anti-*Cryptosporidium* FITC-conjugated. Bars = 10 μm.

Oocysts of *C. muris* CAT21 from the naturally infected cat did not differ morphometrically from those obtained from experimentally infected BALB/c mice (*T*^2^ = 4.32, df_1_ = 2, df_2_ = 24.62, *P* = 0.2208; Table 2). Statistical analysis showed that *C. muris* CAT21 oocysts were not distinguishable in size from *C. muris* RN66 oocysts (*T*^2^ = 12.32, df_1_ = 2, df_2_ = 25.63, *P* = 0.1907), but they were larger than *C. proliferans* oocysts (*T*^2^ = 32.56, df_1_ = 2, df_2_ = 32.27, *P* < 0.0001). Oocysts of *C. muris* isolate CAT21 obtained from a naturally infected domestic cat and from experimentally infected model hosts were detectable in faecal smears using standard staining techniques for the detection of *Cryptosporidium* (Figure 3).

**Table 2.** Morphometric characteristics of oocysts of *C. muris* CAT21, *C. proliferans* and *C. muris* RN66 isolated from different hosts Table 2 long description.

Species/isolate	Host	Length (μm)	Width (μm)	Length/width ratio
*C. muris* CAT21	Cat No. 4	8.43 (7.21–9.55)	6.22 (4.84–7.33)	1.35
*C. muris* CAT21	BALB/c mouse	8.40 (7.48–8.98)	6.16 (5.29–7.16)	1.34
*C. proliferans*	BALB/c mouse	7.77 (6.80–8.80)	5.30 (4.80–6.20)	1.48
*C. muris* RN66	BALB/c mouse	8.41 (7.34–9.23)	6.20 (5.36–7.06)	1.30

### Infectivity and oocyst shedding intensity in experimental hosts

**Figure 4.**
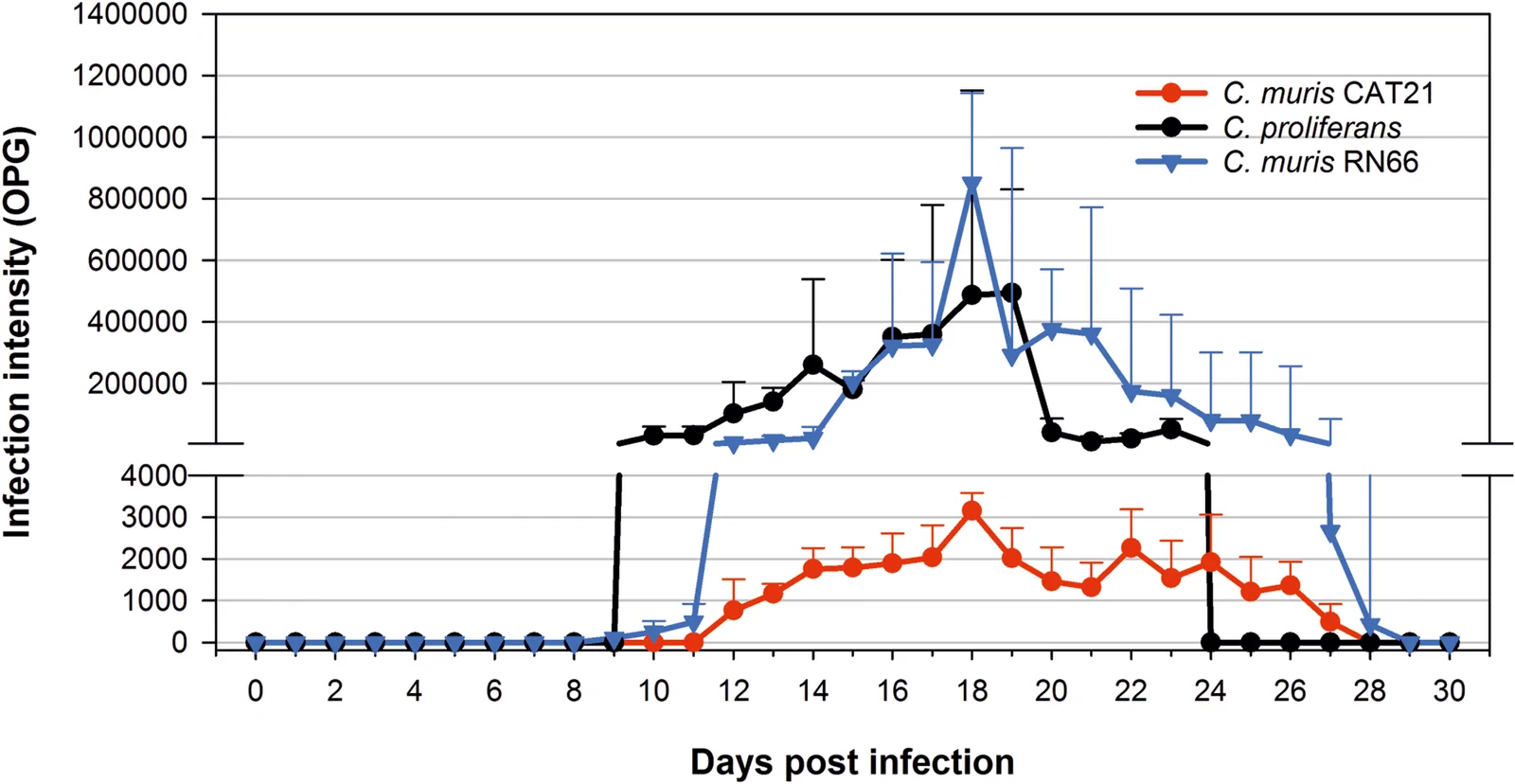
Severity of infection, determined by quantification of oocyst excretion expressed as oocysts per gram (OPG) of faeces, in BALB/c mice infected with *C. muris* CAT21, *C. muris* RN66, or *C. proliferans* at a dose of 1 000 000 oocysts per animal. The course represents the mean values derived from 6 infected animals for each isolate. Figure 4 long description.

Oocyst shedding differed among the tested isolates (Figure 4). The onset of shedding occurred earliest in *C. muris* RN66 (day 9 post-infection), followed by *C. proliferans* (day 10), whereas *C. muris* CAT21 showed the longest prepatent period, with the first detection on day 12 post-infection. The duration of oocyst excretion also varied among isolates. Shedding persisted until days 26–28 in *C. muris* RN66, days 25–27 in *C. muris* CAT21 and days 22–23 in *C. proliferans*. Remarkable differences were observed in shedding intensity. The highest oocyst output was recorded in *C. muris* RN66, reaching a peak of 851 200 OPG. *Cryptosporidium proliferans* showed a lower maximum intensity 493 333 OPG, while *C. muris* CAT21 produced the lowest oocyst counts with a peak 3156 OPG. The total oocyst output (AUC) differed significantly among isolates (*P* = 0.00014). *Cryptosporidium muris* RN66 exhibited the highest total oocyst production (4 080 000 OPG/days), followed by *C. proliferans* (2 770 000 OPG/days), whereas *C. muris* CAT21 showed markedly reduced oocyst output (32 300 OPG/days).

### Degradation of oocysts

**Figure 5.**
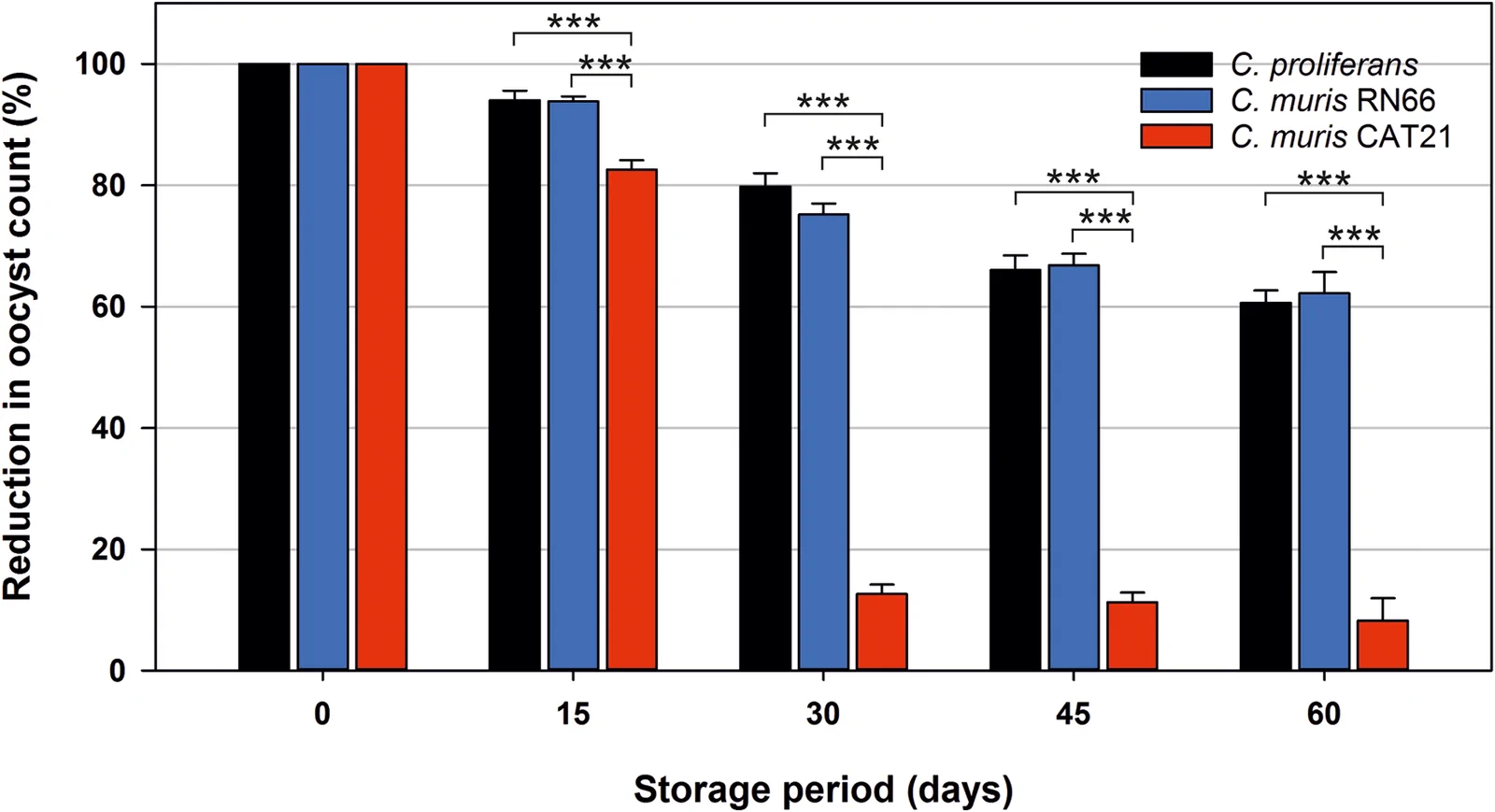
Degradation of oocysts of *C. muris* CAT21, *C. muris* RN66 and *C. proliferans* stored in distilled water at 4°C over a 60-day period, expressed relative to the values measured on day 0. Statistical significance was evaluated as follows: *P* < 0.001 (***). Figure 5 long description.

A significant difference in the loss of oocyst integrity was observed for *C. muris* CAT21 compared with *C. proliferans* and *C. muris* RN66. No significant difference in oocyst integrity was detected between *C. proliferans* and *C. muris* RN66 during the 60-day observation period, with a decrease in oocyst numbers of approximately 35–40%. In contrast, *C. muris* CAT21 showed a marked decline in oocyst numbers during storage already after 15 days (−20%), followed by a sharp decrease by day 30 (−90%; Figure 5).

## Discussion

In this study, we describe a natural infection of a domestic cat caused by *C. muris* CAT21, manifested by a relatively high intensity of infection for more than 90 days, but with production of oocysts displaying phenotypic properties different from those expected for this *Cryptosporidium* species. The study therefore does not represent merely another host record, but primarily a biologically unusual interaction between a parasite and a non-specific host, suggesting an altered course of parasite development.

The typical hosts of *C. muris* are rodents, especially mice and rats, in which the parasite produces stable infections characterized by relatively high oocyst shedding and a patent period usually lasting several weeks, with typical tissue localization in the gastric glands (Iseki et al., 1989; Kváč et al., 2011). Although *C. muris* has also been repeatedly recorded in other mammalian hosts, including humans, non-human primates, other rodents, ruminants, rabbits, seals, dogs or cats, described infections outside rodent hosts are rare and the biological properties of such isolates remain poorly explored (Iseki et al., 1989; Gatei et al., 2002; Warren et al., 2003; Pavlásek and Ryan, 2007; Lupo et al., 2008; Feng, 2010; Kodádková et al., 2010; Kváč et al., 2016).

Iseki et al. (1989) experimentally demonstrated that cats are susceptible to *C. muris* infection. However, only a few cases of natural detection of oocysts or specific *C. muris* DNA in cats have been described to date (Table 1). Only in 3 of them, an active infection can be stated with certainty, based either on histological findings in a gastric biopsy or on long-term monitoring of parasite shedding in faeces (Iseki et al., 1989; Pavlásek and Ryan, 2007; FitzGerald et al., 2011), which excludes mechanical transport of oocysts from food (infected rodents). Detection of parasite-specific DNA in an atypical host during a single examination, without evidence of developmental stages, is often interpreted in the literature as passive passage of oocysts after ingestion of the parasite’s natural host, serving as a prey (Xiao et al., 2004; Ng et al., 2006; Kváč et al., 2012). In our case, active infection was demonstrated by a combination of several independent findings. The monitored cat shed oocysts daily for more than 90 days, which is incompatible with simple mechanical passage through the digestive tract after ingestion of infected prey. Active infection was further confirmed by histological detection of developmental stages in the gastric mucosa. Together, these findings reliably document completion of the parasite life cycle in the feline host.

Molecular analysis of 5 selected loci showed 100% identity between the isolate obtained from the cat and isolates originating from mice. Despite identical sequences at all analysed genetic loci, and while acknowledging that genome-wide differences cannot be completely excluded, oocysts obtained from the feline host exhibited distinct biological properties. The morphometry and morphology of *C. muris* CAT21 oocysts obtained from the cat and from experimentally infected rodents were indistinguishable. This observation is consistent with previous studies showing that oocysts of *Cryptosporidium* spp. retain their characteristic shape and size after passing through different host species (Holubová et al., 2016; Ježková et al., 2021). A marked difference was observed in oocyst integrity during storage, which directly affected survival in the external environment. Most oocysts of the *C. muris* CAT21 isolate lost integrity/spontaneously excysted already between days 15–30 of storage, which is not typical for mammalian gastric cryptosporidia. Our comparative observations with *C. proliferans* and *C. muris* RN66 (reference strain), as well as previously published data on *C. andersoni*, show that oocysts of these species retain integrity for several months under comparable conditions (Kváč et al., 2007).

Consistent with the results of Pavlásek and Ryan (2007), we demonstrated that *C. muris* oocysts originating from a naturally infected cat retain their infectivity and are capable of infecting experimental mice. However, the course of infection differed in dynamics compared with the reference isolate *C. muris* RN66. While neither the prepatent nor patent period differed significantly, the *C. muris* CAT21 isolate showed several orders of magnitude lower oocyst shedding intensity. These results indicate that parasite development in the feline host did not lead to a loss of viability but to a reduction in the ability to transmit oocysts. The parasite was able to complete its life cycle yet produced a transmissible stage with a limited capacity to infect subsequent hosts.

Reduction of transmission fitness after development in a non-specific/non-optimal host is well documented in other members of the phylum Apicomplexa. In *Toxoplasma gondii*, adaptation of isolates to cell culture and repeated passage led to marked phenotypic changes, including reduced ability to form tissue cysts and altered virulence in experimental mice (Villard et al., 1997; Colos-arango et al., 2023). Similarly, in *Babesia bovis*, repeated passage in an immunologically non-optimal host selects populations with reduced virulence, while retaining the capacity for invasion and multiplication (Callow and Stewart, 1978). In coccidia of the genus *Eimeria*, selection of so-called precocious (attenuated) lines leads to phenotypic changes. These lines complete their life cycle and produce morphologically normal and viable oocysts, but exhibit reduced reproductive capacity and lower infectivity in subsequent hosts (Richards et al., 2006; McDonald and Shirley, 2009). Our observations in *C. muris* CAT21 correspond to this biological principle, according to which the parasite remains infectious and able to complete development, but the properties of its transmissive stages and subsequent infectivity may change substantially.

From a clinical perspective, infection in the monitored cat proceeded without apparent symptoms, consistent with the results of experimental infection of a cat with the *C. muris* RN66 isolate (Iseki et al., 1989) and the general fact that infection with gastric cryptosporidia of mammals is usually asymptomatic (Rhee et al., 1991; Ozkul and Aydin, 1994; Hikosaka and Nakai, 2005; Kváč et al., 2008). In contrast, Pavlásek and Ryan (2007) described cats with *C. muris* infection showing marked clinical signs including diarrhoea and vomiting. The authors attributed these signs to *C. muris* infection but completely overlooked that the cat was simultaneously infected with the roundworm *Toxocara cati*. It is therefore likely that the health problems in that cat were caused by *T. cati* infection, for which the described clinical signs are typical (Overgaauw, 1997; Ursache et al., 2021). FitzGerald et al. (2011) also described clinical signs associated with mixed infection by *C. muris* and *C. felis*, and noted that it is difficult to attribute the clinical presentations to either species.

## Conclusions

The results of our study, in agreement with previous observations, confirm that the cat may serve as a susceptible but epidemiologically inefficient host of *C. muris*. Although long-term oocyst shedding occurs in cats (Iseki et al., 1989; FitzGerald et al., 2011), their reduced infectivity to the natural rodent host indicates a limited capacity for further transmission (this study). The cat therefore probably does not represent an important infection reservoir. The mere presence of oocysts in faeces does not necessarily reflect the epidemiological importance of a host, because the quality of the transmissive stage may be strongly influenced by the host environment in which it was produced. The precise reason why only some hosts show susceptibility to infection by atypical parasites remains unclear. Exposure alone is probably insufficient, as other cats from the same environment remained uninfected. Host factors (individual immune response, physiological conditions of the gastrointestinal tract) likely play a role, although an influence of the isolate itself, such as its adaptation state or quality of transmissive stages, cannot be excluded. Occurrence of *C. muris* in atypical hosts may thus reflect a complex host–parasite interaction, the mechanism of which remains a subject for further research. Elucidating these mechanisms may also help explain why human infections with *C. muris* remain exceptionally rare, despite the parasite’s ability to infect humans (Chappell et al., 2015), and may enhance our understanding of cross-species transmission within a One Health framework.

## Data Availability

All data are available in the publication. Raw data may be obtained from the Institute of Parasitology, Biology Centre, Czech Academy of Sciences, České Budějovice, Czech Republic.

## Table 1 Long description

Columns list year of detection, type of infection, number of cats examined, diagnostic method, evidence of oocyst infectivity, sampling strategy, and reference. In 1989, an experimental infection examined 3 cats, used microscopy, reported infectivity evidence as yes in mice, and used repeated sampling, reference Iseki et al., 1989. In 2006, a natural infection examined 1 cat, used PCR, reported no infectivity evidence, and used one shot sampling, reference Santín et al., 2006. In 2007, the type of infection cell contains 1, the number of cats examined cell is blank, diagnostic method lists microscopy and PCR, infectivity evidence is yes in mice, and sampling strategy is repeated sampling, reference Pavlásek and Ryan, 2007. In 2011, the type of infection cell contains 1, the number of cats examined cell is blank, diagnostic method lists histology and PCR, infectivity evidence is no, and sampling strategy is repeated sampling, reference FitzGerald et al., 2011. In 2015 and 2025, the type of infection cell contains 1, the number of cats examined cell is blank, diagnostic method is PCR, infectivity evidence is no, and sampling strategy is one shot sampling, references Yang et al., 2015 and Barrios et al., 2025.

Navigate back to Table 1.

## Figure 4 Long description

The y-axis is labeled, Infection intensity (OPG). The x-axis is labeled, Days post infection. The x-axis runs from 0 to 30 with tick marks every 2. The y-axis is split into two ranges. The lower range runs from 0 to 4000 with tick marks every 1000. The upper range runs from 0 to 1 400 000 with tick marks every 200 000. A legend at the upper right lists three series: C. muris CAT21, C. proliferans and C. muris RN66. Cryptosporidium muris CAT21 is shown with circular markers connected by a line. Values are at 0 from day 0 through day 11. At day 12 the value is about 500. Day 13 about 1 000. Day 14 about 1500. Day 15 about 1 500. Day 16 about 1 700. Day 17 about 1 700. Day 18 about 2 000. Day 19 about 3000. Day 20 about 2 000. Day 21 about 1 500. Day 22 about 1 500. Day 23 about 2 500. Day 24 about 1 500. Day 25 about 2 000. Day 26 about 1 000. Day 27 about 500. Day 28 about 0. Day 29 about 0. Day 30 about 0. Cryptosporidium proliferans is shown with circular markers connected by a line. Values are at 0 from day 0 through day 9. At day 10 the value is about 100 000. Day 11 about 100 000. Day 12 about 150 000. Day 13 about 200 000. Day 14 about 300 000. Day 15 about 200 000. Day 16 about 350 000. Day 17 about 400 000. Day 18 about 450 000. Day 19 about 500 000. Day 20 about 100 000. Day 21 about 100 000. Day 22 about 100 000. Day 23 about 120 000. Day 24 about 0. Day 25 about 0. Day 26 about 0. Day 27 about 0. Day 28 about 0. Day 29 about 0. Day 30 about 0. Cryptosporidium muris RN66 is shown with downward triangle markers connected by a line. Values are at 0 from day 0 through day 11. At day 12 the value is about 100 000. Day 13 about 100 000. Day 14 about 200 000. Day 15 about 300 000. Day 16 about 350 000. Day 17 about 400 000. Day 18 about 850 000. Day 19 about 350 000. Day 20 about 400 000. Day 21 about 350 000. Day 22 about 350 000. Day 23 about 250 000. Day 24 about 200 000. Day 25 about 200 000. Day 26 about 150 000. Day 27 about 150 000. Day 28 about 0. Day 29 about 0. Day 30 about 0.

Navigate back to Figure 4.

## Figure 5 Long description

The bar graph has a legend with three series, C. proliferans, C. muris RN66 and C. muris CAT21. The x axis label is Storage period left parenthesis days right parenthesis, with tick labels 0, 15, 30, 45, 60. The y axis label is Reduction in oocyst count left parenthesis percent right parenthesis, with tick labels 0, 20, 40, 60, 80, 100. At 0 days, C. proliferans 100 percent, C. muris RN66 100 percent, C. muris CAT21 100 percent. At 15 days, C. proliferans about 95 percent, C. muris RN66 about 90 percent, C. muris CAT21 about 80 percent. A bracket above the 15 day group shows * * asterisk. At 30 days, C. proliferans about 80 percent, C. muris RN66 about 75 percent, C. muris CAT21 about 12 percent. Brackets above the 30 day group show * * asterisk. At 45 days, C. proliferans about 65 percent, C. muris RN66 about 70 percent, C. muris CAT21 about 12 percent. Brackets above the 45 day group show * * asterisk. At 60 days, C. proliferans about 60 percent, C. muris RN66 about 62 percent, C. muris CAT21 about 8 percent. Brackets above the 60 day group show * * asterisk.

Navigate back to Figure 5.

## Table 2 Long description

Morphometric measurements are listed for oocysts by species or isolate and host, with columns for length in micrometers, width in micrometers, and length to width ratio. Cryptosporidium muris CAT21 from Cat No. 4 has length 8.43 with range 7.21 to 9.55, width 6.22 with range 4.84 to 7.33, and ratio 1.35. Cryptosporidium muris CAT21 from BALB/c mouse has length 8.40 with range 7.48 to 8.98, width 6.16 with range 5.29 to 7.16, and ratio 1.34. Cryptosporidium proliferans from BALB/c mouse has length 7.77 with range 6.80 to 8.80, width 5.30 with range 4.80 to 6.20, and ratio 1.48. Cryptosporidium muris RN66 from BALB/c mouse has length 8.41 with range 7.34 to 9.23, width 6.20 with range 5.36 to 7.06, and ratio 1.30.

Navigate back to Table 2.
